# Intelligence

**Published:** 1807-12

**Authors:** 


### INTELLIGENCE.

M. Caselli, a celebrated Neapolitan aftronomer, accounts for the extraordinary heat of the paft fummer, which continued fo intenfe throughout the whole month of September, by afcribing it to the extreme purity of the face of the fun, which this year was turned towards the earth. For near two months he obferved none of the fpots which are commonly perceived in it. Hence M. Cafelli concludes, that the fun's rays having been emitted in greater abundance, and with lefs interruption, produced that violent degree of heat, which, though it scorched the fields, neverthelefs increafed their fertility.

Mr. John Sadler, of Newcaftle upon Tyne, has announced the publication of an Encyclopœdia of Manufactures, in which it is intended to trace every raw material from its growth, till it be delivered into the hands of the workman. The various modes of fabrication; the improvements which each art has received; and the hiftory and progrefs of thefe improvements will be fully detailed, accompanied by hints for their farther extenfion and fimplification.

M. Griebel, clock-maker, at Paris, has invented a clock without weights, of a globular form, of which the dial plate is transparent, and by means of a reflecting lamp on Argand's construction, shews the figure to a great diftance. By a particularity of formation, neither the wheels, the hands, nor the pendulum, cast any shadow. The light may be made stronger or weaker, and adapted to the sick chamber, or to clocks in the most public situations, where it answers the purpose of a time-piece and of a lamp at the same time.

A large

A large impression of Dr. TROTTER's *View of the Nervous Temperament*, having met a most rapid sale in London, an improved edition of that Work is now in the Press, and will be published in the present Year.

Mr. WISHART, Fellow of the Royal College of Surgeons, Edinburgh, has in the prefs a Tranflation of Prof. Scarpa's fplendid work on Aneurifm, with Notes.

Dr. R. ROBERTSON, intends to publifh in two volumes octavo, A View of the Natural Hiftory of the Atmofphere, and its Influence in the Sciences of Medicine and Agriculture, including an Effay on Contagion.

Dr. JAMES SAUNDERS, of Edinburgh, has in the prefs, A Treatife on Pulmonary Confumption; with an Enquiry concerning the Foxglove.

The fame gentleman will shortly publifh An Enquiry concerning the Accumulation of Water in the Brain, called Hydrocephalus; in which he will endeavour to prove, that it can be either prevented or cured with as much facility as any other of the more dangerous difeafes.

An Effay on the Pathology of the Human Eye, by Mr. JAMES WARDROP, Fellow of the Royal College of Surgeons, Edinburgh, is in preparation. The various morbid appearances of the eye will be illuftrated by coloured engravings, after drawings by Mr. SYME.

Mr. PARKINSON has nearly ready for publication the fecond volume of his work on the Organic Remains of the former World.

Mr. PROUSS purpofes to publifh, in the courfe of the winter, Phyfiological Effays on Infanity, with Reflections and Analytical Refearches relative to the Circumftances which predifpofe the Mind to that Difeafe, and which caufe and continue it.

Mr. ALEXANDER WILSON, of Philadelphia, is printing the Ornithology of America, or the Natural Hiftory of the Birds of the United States.

